# Supplementary material for: Randomised controlled trial comparing hydroxyapatite coated uncemented hemiarthroplasty with cemented hemiarthroplasty for the treatment of displaced intracapsular hip fractures: a protocol for the WHITE 5 study
Source: BMJ Open. 2019 Dec 9;9(12):e033957. doi: 10.1136/bmjopen-2019-033957 (PMC6924810; doi:10.1136/bmjopen-2019-033957)
Supplement: Author's manuscript [file bmjopen-2019-033957.draft_revisions.pdf]

BMJ Open

BMJ Open is committed to open peer review. As part of this commitment we make the peer review history of every article we publish publicly available.

When an article is published we post the peer reviewers' comments and the authors' responses online. We also post the versions of the paper that were used during peer review. These are the versions that the peer review comments apply to.

The versions of the paper that follow are the versions that were submitted during the peer review process. They are not the versions of record or the final published versions. They should not be cited or distributed as the published version of this manuscript.

BMJ Open is an open access journal and the full, final, typeset and author-corrected version of record of the manuscript is available on our site with no access controls, subscription charges or pay-per-view fees (http://bmjopen.bmj.com).

If you have any questions on BMJ Open's open peer review process please email info.bmjopen@bmj.com

BMJ Open

Randomised controlled trial comparing hydroxyapatite coated uncemented hemiarthroplasty with cemented hemiarthroplasty for the treatment of displaced intracapsular hip fractures: a protocol for the WHITE 5 study

Journal:	*BMJ Open*
Manuscript ID	bmjopen-2019-033957
Article Type:	Protocol
Date Submitted by the Author:	29-Aug-2019
Complete List of Authors:	Fernandez, Miguel; University of Oxford Nuffield Department of Orthopaedics Rheumatology and Musculoskeletal Sciences, Oxford Trauma; University Hospitals Coventry and Warwickshire NHS Trust, Trauma & Orthopaedic Surgery<br>Achten, Juul; University of Oxford, NDORMS<br>Lerner, Robin; University of Oxford Nuffield Department of Orthopaedics Rheumatology and Musculoskeletal Sciences, Oxford Trauma<br>Mironov, Katy; University of Oxford Nuffield Department of Orthopaedics Rheumatology and Musculoskeletal Sciences, Oxford Trauma<br>Parsons, Nicholas; University of Warwick, Warwick Medical School<br>Dritsaki, Melina; University of Warwick,<br>Png, May ee; University of Oxford, NDORMS<br>McGibbon, Alwin; Patient representative, UNTRAP University of Warwick<br>Gould, Jenny; Patient representative<br>Griffin, Xavier; University of Oxford Nuffield Department of Orthopaedics Rheumatology and Musculoskeletal Sciences, Kadoorie Centre<br>Costa, Matthew; University of Oxford, Nuffield Department of Orthopaedics, Rheumatology, and Musculoskeletal Sciences
Keywords:	hip fracture, randomised controlled trial, hemiarthroplasty

SCHOLARONE™
Manuscripts

Randomised controlled trial comparing hydroxyapatite coated uncemented hemiarthroplasty with cemented hemiarthroplasty for the treatment of displaced intracapsular hip fractures: a protocol for the WHITE 5 study

M. A. Fernandez[1,2], J. Achten[1], R. G. Lerner[1], K. Mironov[1], N. Parsons[3], M. Dritsaki[4], M. E. Png[4], A. McGibbon[5], J. Gould[5], X. L. Griffin[1], and M. L. Costa[1*]

[1]Oxford Trauma, NDORMS, University of Oxford, Oxford OX3 9DU, UK

[2]University Hospitals Coventry & Warwickshire NHS Trust, Clifford Bridge Road, Coventry, CV2 2DX, UK

[3]Statistics and epidemiology unit, Warwick Medical School, University of Warwick, Gibbet Hill Road, Coventry, CV4 7AL, UK

[4]Oxford Clinical Trial Unit, NDORMS, University of Oxford, Oxford OX3 9DU, UK

[5]Patient representatives

*corresponding author: Prof Matthew Costa, Oxford Trauma, NDORMS, Kadoorie Centre, John Radcliffe Hospital, Headley Way, Oxford OX3 9DU, UK. Matthew.costa@ndorms.ox.ac.uk

Word count
4567

Key words
Hip fracture, hemiarthroplasty, randomised controlled trial

For peer review only

ABSTRACT

Introduction

Hip fracture is a serious injury in adults, especially those aged over 60 years. The most common type of hip fracture (displaced intracapsular) is treated for the majority of patients with a partial hip replacement (hemiarthroplasty). The hemiarthroplasty implant can be fixed to the bone with or without bone cement. Cement is the current recommended technique but recently some risks have been identified which could potentially be avoided by using uncemented implants. Controversy therefore remains about which type of hemiarthroplasty offers patients the best outcomes.

This is the protocol for a multicentre randomised controlled trial comparing cemented hemiarthroplasty versus uncemented hemiarthroplasty for patients 60 years and over with a displaced intracapsular hip fracture.

Methods and analysis

Multi-centre (a minimum of 7 UK hospitals), multi-surgeon, parallel group, two-arm, superiority, randomised controlled trial. Patients aged 60 years and older with a displaced intracapsular hip fracture treated with hemiarthroplasty surgery are eligible. Participants will be randomly allocated on a 1:1 basis to either a cemented hemiarthroplasty or a modern hydroxyapatite coated uncemented hemiarthroplasty. Otherwise all care will be in accordance with the National Institute for Health and Care Excellence guidance. A minimum of 1128 patients will be recruited to obtain 90% power to detect a 0.075-point difference in the primary endpoint: health-related quality of life (EuroQol 5 Dimensions 5 Levels EQ-5D-5L) at 4-months post-injury. The treatment effect will be estimated using a two-sided t-test adjusted for age, gender and cognitive impairment based on an intention-to-treat analysis. Secondary outcomes include mortality, complications including revision surgery and cause, mobility status, residential status, health-related quality of life at 1 and 12-months and health resource use. A within-trial economic analysis will be conducted.

Ethics, dissemination and funding

Wales Research Ethics Committee 5 approved the feasibility phase on 2nd December 2016 (16/WA/0351) and the definitive trial on 22nd November 2017 (17/WA/0383). This study is sponsored by the University of Oxford and funded by the National Institute for Health Research, Research for Patient Benefit (PB-PG-0215-36043 and PB-PG-1216-20021). A manuscript for a peer-reviewed journal will be prepared and the results shared with patients via local mechanisms at participating centres.

Trial Registration number: ISRCTN18393176

Strengths & Limitations

- Pragmatic multicentre randomised controlled trial
- Powered to detect differences in health-related quality of life
- Inclusion of participants with and without cognitive impairment
- Outcomes include the UK core outcome set for hip fracture

For peer review only

- The trial will not capture late complications, implant failure or revision surgery beyond 1-year post injury

INTRODUCTION

Hip fracture is one of the biggest challenges facing patients and healthcare systems. Every year in the UK there are more than 65,000 hip fractures.[1] Displaced 'intracapsular' fractures represent approximately half of all hip fractures. In these fractures, the head of the femur is broken off at the neck. The blood supply to the femoral head is tenuous and, even if the fracture is fixed back in to its anatomical position, fracture healing is unreliable and 40% of patients will have a failure of fixation. For this reason, arthroplasty (replacement) of the femoral head is preferred in the UK. A minority of patients receive a 'total hip arthroplasty' (replacing the 'socket' of the hip joint as well as the femoral head) if they are particularly active before the fracture, but in most cases only the head of the femur is replaced; a hemiarthroplasty.

The National Institute for Health and Care Excellence (NICE)[2] guidelines for hip fracture recommend the use of bone cement to aid in the fixation of the hemiarthroplasty implant to the bone. These guidelines are based on outcomes of studies which show less postoperative pain and better function when cemented components were used compared to their first generation uncemented counterparts (e.g. the Austin Moore prosthesis). The evidence base for these guidelines are summarised in a Cochrane review[3] which acknowledged the lack of evidence for more contemporary uncemented implants and concluded that "there is still a place for trials of contemporary uncemented stems with cemented stem hemiarthroplasty". A recent independent systematic review and meta-analysis compared cemented hemiarthroplasty with modern uncemented hemiarthroplasty and concluded: *"there remains a need for a methodologically sound, large muticentre RCT comparing modern cemented and cementless hemiarthroplasty stems in the medium – and long-term, not only focusing on mortality and complications but also on patient reported outcome measures."*[4]

This is the protocol for a multi-centre parallel-group randomised controlled trial, that compares cemented hemiarthroplasty and modern hydroxyapatite coated uncemented hemiarthroplasty.

AIMS AND OBJECTIVES

The aim of this randomised controlled trial is to compare health-related quality of life (HRQoL) in participants over 60 years of age with a displaced intracapsular hip fracture receiving a modern hydroxyapatite coated uncemented hemiarthroplasty versus the current standard-of-care cemented hemiarthroplasty.

The primary objective is:

- To quantify and draw inferences on observed differences in participants' HRQoL between the trial treatment groups at 4 months post-injury.

The secondary objectives are:

For peer review only

- To quantify and draw inferences on the observed differences in participants' HRQoL between the trial treatment groups at 1 and 12 months post-injury
- To quantify and draw inferences on the observed differences in the proportion of complications, including further surgery, within the first 12 months post-injury between the trial treatment groups
- To quantify and draw inferences on observed differences in mortality, mobility and residential status at 1, 4 and 12 months post-injury
- To investigate, using appropriate statistical and economic analytical methods, the resource use, costs and incremental cost effectiveness of the trial treatment groups at four months post-injury

METHODS AND ANALYSIS

Study design

A multi-centre, parallel group, two-arm, superiority, standard-of-care randomised controlled superiority trial in a minimum of 7 UK recruitment centres. Each of these recruitment centres is recruiting hip fracture patients as part of the World Hip Trauma Evaluation (WHiTE) hip fracture cohort study.[5] The study is conducted in two phases: an initial feasibility phase in which the acceptability of the interventions and trial processes were tested, and a definitive phase which comprises the main trial. Data from the feasibility phase will be locked, and not analysed, at completion. At the end of the definitive main trial phase, data from the two phases will be analysed together as a single dataset.

Trial Registration

Application for trial registration was made to the ISRCTN on 7th March 2017 prior to commencing patient enrolment.

Patient and Public Involvement

At the centre of this work is the potential for patient benefit by reducing the risks of hip fracture surgery and improving patient outcomes. The involvement of patient and public representatives has therefore been a key element of this work to ensure that the running of the trial remains patient focused. JG and AM are patient representative coauthors who have been involved in all aspects of this work from designing the trial, obtaining grant funding, developing the trial materials, and participation in the running of the trial. More specifically, the role of patient representatives has been to ensure that trial processes and information are focused on patients, to provide the patient perspective as members of the trial management group, and to ensure that all trial processes have taken into account the needs of patients both with and without cognitive impairment.

Eligibility

Patients will be screened against the following criteria:

Inclusion criteria

- All patients, both those with and without capacity, presenting with a displaced intracapsular fracture of the hip suitable for hemiarthroplasty.

For peer review only

Exclusion criteria

- Patients younger than 60 years of age
- Patients who are managed non-operatively
- Patients who are treated with a total hip replacement

Consent

Patients with a hip fracture are a clinical priority for urgent operative care. They undergo surgery on the next available trauma operating list. All patients with a fracture of the hip are in pain and will have received opiate analgesia. It is therefore understandable that the majority of patients find the initial period of their treatment in hospital confusing and disorientating. Similarly, patients' next of kin, carers and friends are often anxious at this time and may have difficulty in absorbing the large amounts of information that they are given about the injury and plan for treatment. In this emergency situation the focus is on obtaining consent for surgery (where possible) and on informing the patient and any next of kin about immediate clinical care. It is often not possible for the patient, relative or carer (consultee) to review trial documentation, consider the information and communicate an informed decision about whether they would wish to participate in the study. The consent procedure for this trial will reflect that of the surgery, with the clinical team assessing capacity before taking consent for the surgical procedure, and this capacity assessment then being used to guide the proper approach to consenting to the research. An appropriate method, in line with the mental capacity act and approved by the National Research Ethics Committee, will be used to gain either prospective or retrospective consent from the patient or appropriate consultee by a Good Clinical Practice (GCP)-trained, appropriately delegated member of the research team.

Randomisation and blinding

The allocation sequence will be computer generated by the trial statistician and administered, using secure online randomisation, by Oxford Clinical Trials Research Unit (OCTRU), University of Oxford. Participants will be enrolled by the operating surgeon or trial research associate. The research associate will receive notification of the allocated treatment and inform the surgeon and the operating theatre staff. The randomisation algorithm will use variable block sizes (2, 4 and 6), to ensure good treatment balance, at each of the recruitment centres. The surgery will be performed under the care of the consultant surgeon on-call at the time the patient is admitted as per local practice in the recruitment centres. The large number of surgeons – previous experience in similar trials suggests over 200 surgeons will take part – and the wide skill mix should minimise any surgeon-specific effects such that stratification by surgeon is unnecessary. In order to negate bias in the self-reported HRQoL outcome measures participants will be blinded to treatment allocation. The operating surgeon cannot be blinded to the allocation but they will not be involved in the assessment of outcomes. Patients will be blinded until the completion of the trial when the blinding will be broken if requested by the patients. There will be no formal analysis of the success of the blinding.

For peer review only

Post-randomisation withdrawals and exclusion

Throughout the study, screening logs will be kept to determine the number of patients assessed for eligibility and reasons for any exclusion. Participants may withdraw from the study at any time without prejudice. If a participant withdraws from the study completely, data collected up until the point of withdrawal will be included in the final analysis. Patients who decline to continue to take part will be given the opportunity to discuss and inform the research team of the reasoning behind their decision not to take part. Similarly, data obtained from participants who die before they or a personal consultee has been approached about continued consent/agreement will be included in the final analysis.

Treatments

Standardised treatment pathway

Participants will usually be assessed in the Emergency Department. Diagnosis of a hip fracture will be confirmed by a plain radiograph, as per routine clinical care. Supplementary imaging will be at the discretion of the treating clinical team. Routine investigations, anaesthetic assessment, antibiotic and venous thromboembolic prophylaxis will be used as per local policy. A regional or general anaesthesia technique will be used for every participant as per routine clinical care. All participants will receive perioperative prophylactic antibiotics in accordance with current protocols agreed at each recruitment centre. Postoperative analgesia will be prescribed intra-operatively and reviewed by the responsible clinical teams as appropriate.

In the postoperative period, as per standard of care, all participants will undergo a physiotherapy and occupational therapy assessment to create a rehabilitation plan. The aim of this plan will be for participants to mobilise through early, active, full weight bearing. Participants will be discharged from the acute Orthopaedic Trauma Ward at the earliest safe opportunity to the most appropriate discharge destination as determined by the multi-disciplinary clinical team. Participants will be given the relevant NHS Trust information packs.

Interventions

Appropriate preparation, positioning and surgical technique will be left to the discretion of the operating surgeon, as per their normal clinical practice. Participants will be randomly allocated to one of two treatment groups:

Group 1: Cemented hemiarthroplasty. Replacement of the femoral head and neck with a cemented femoral stem and head as per NICE guidance; the current standard-of-care (control) intervention.

Group 2: Modern uncemented hemiarthroplasty. Replacement of the femoral head and neck with a modern (hydroxyapatite coated) uncemented hemiarthroplasty; the alternative intervention.

Both types of hip replacement are used routinely throughout the NHS. While the principles of both cemented and uncemented arthroplasty are inherent in the

For peer review only

technique, the surgical approach and other technical aspects of the surgery will be left to the discretion of the senior operating surgeon as per their normal practice. This will ensure that the results of this pragmatic trial can be extrapolated broadly across the NHS.

Outcomes and Data Management

Personal data collected during the study will be handled and stored in accordance with the 1998 Data Protection Act, which requires data to be anonymised as soon as it is practical to do so. The data collected from participants will be entered in linked-anonymised form to the trial database. All electronic patient-identifiable information will be stored on a secure, password-protected database at the University of Oxford, accessible only to the research team. Any paper copies of identifiable data, and corresponding re-identifying links to the participant trial ID, will be stored separately, in a locked cabinet in an access-restricted part of the Kadoorie Centre, John Radcliffe Hospital, Oxford. The study databases will be developed by the trial programmer and all specifications agreed between the trial programmer, statistician and trial manager and other relevant members of the trial team. The procedure for data entry will be documented in the data management plan. As per routine clinical care, the existing National Hip Fracture Database dataset will be collected via telephone interview or postal questionnaire. We will send data collected at follow up to the NHFD via a secure email account for them to upload using the participant's date of birth and NHS number as identifiers.

Primary outcome Measure

The study primary outcome measure is EuroQol 5 Dimensions 5 Levels (EQ-5D-5L) index score at 4-months post-injury. The index score is derived from the 5 health state domains using the crosswalk value data sets.[6] EQ-5D-5L is a validated, self-rated instrument comprising a visual analogue scale (VAS) measuring health and a health status instrument, consisting of a five-level response (no problems, some problems, moderate problems, severe problems and unable) for five domains related to daily activities[7]; (i) mobility, (ii) self-care, (iii) usual activities, (iv) pain and discomfort and (v) anxiety and depression. The EQ-5D-5L instrument [7] facilitates the generation of a utility score from a person's health related quality of life, which refers to the preference that individuals have for any particular set of health outcomes. As per the current NICE position statement, the responses to the EQ-5D-5L will be converted into multi-attribute utility scores using an approved "cross-walk" to the 3L instrument and its established time trade-off utility algorithm for the UK population.[8]. A respondent's EQ-VAS gives self-rated health on a scale where the endpoints are labelled 'best imaginable health state' (100) and 'worst imaginable health state' (0). EQ-5D is part of the UK Core outcome set[9] and has been shown to be responsive to change,[10,11] including when reported by proxy for those with cognitive impairment.[12,13]

Secondary outcome measures

HRQoL (*EQ-5D-5L)* at 1 and 12 months post-injury. Mortality, revision surgery and cause, and all complications will be obtained from the patients' medical record. Patient interviews and questionnaires will be used to assess mobility status (walking

For peer review only

ability indoors and outdoors) and residential status. An assessment of resource use will inform the economic analysis plan below.

Sample size

The best available evidence we have from data collected during previously performed studies in this patient population suggests that the standard deviation for EQ-5D-5L at 4 months post-injury is approximately 0.3 points.[10] The best available evidence for what constitutes a minimal important difference (MID) for EQ-5D comes from a review of MID estimates.[14] A review of the literature shows an estimated median value of 0.08 for the MID for EQ-5D. Using our previously established standard deviation (SD=0.33), this suggests a standardised effect size of approximately 0.24; a 'small to moderate effect' based on Cohen's criteria.[15] Assuming that the EQ-5D at 4 months post-injury has an approximate normal distribution, which Parsons et al[10] suggests is reasonable, and a 1:1 allocation ratio, then if the true difference between the experimental and control group EQ-5D means is in the range 0.07 to 0.08, we will need to recruit the below number of participants (see Table 1) in each group to be able to reject the null hypothesis that the population means are equal with probability (power) 0.8 and 0.9 and type I error rate of 5% (significance).

Taking the intermediate MCID of 0.075, for 80% (90%) power, we would need to recruit 253 (338) patients in both the experimental arm and in the control arm, 506 (676) in total. In this population we expect considerable loss to follow-up due mainly to patients declining consent to further follow-up and incapacity, so we have assumed that only 60% of recruited study participants will be available at the definitive endpoint at 4 months. This gives a total sample size of 844 for 80% power and 1128 for 90% power. Conservatively, we aim to recruit 1128 to ensure 90% power based upon these assumptions.

Table 1: Group sizes required following sample size calculations.

**Power**	**MCID**		
	**0.07**	**0.075**	**0.08**
**80%**	290	253	222
**90%**	387	338	297

Statistical analysis

The main analysis will investigate differences in the primary outcome measure, EQ-5D (Health Related Quality of Life; HRQoL), between the two treatment groups on an intention-to-treat (ITT) basis at 4 months post-injury on a complete case basis. The 'death-adjusted' ED-5D measure, imputing death as zero, is the preferred analysis option in this population, and will be used for all analyses.[16] An initial analysis will test for differences between treatment groups using linear regression analysis, based on a Normal approximation for EQ-5D. Tests will be two-sided and considered to provide evidence for a significant difference if p-values are less than 0.05 (5% significance level). Estimates of treatment effects will be presented with 95% confidence intervals.

For peer review only

The stratification used for randomization will ensure balance in treatment allocation across recruitment centres. In addition to the unadjusted analyses we will also undertake regression analyses to adjust for any imbalance between treatment groups in patient baseline (pre-injury) EQ-5D, age and gender. Age and gender are likely to be reported by all study participants, however if baseline EQ-5D is poorly reported, such that it reduces the available sample size for the definitive analysis, then it will not be included in the definitive model. The fixed effects analysis (linear regression model) will be generalized by adding a random effect for recruitment centre to allow for possible heterogeneity in patient outcomes due more generally to the recruitment centre. The adjusted mixed-effects regression will be the definitive (primary) analyses and will be reported as such. Analyses will be undertaken using the specialist mixed-effects modelling functions available in the software package R (http://www.r-project.org/).[17] EQ-5D data will be assumed to be approximately normally distributed; possibly after appropriate variance-stabilising transformation. The primary focus will be the comparison of the two treatment groups of patients, and this will be reflected in the analysis which will be reported together with appropriate diagnostic plots that check the underlying model assumptions. Results will be presented as mean differences between the trial groups, with 95% confidence intervals. The primary analysis will be on an ITT basis. However, a per-protocol (PP) analysis will also be undertaken by repeating the primary outcome analysis using the actual treatment received by participants (in contrast to the allocated treatment) as a sensitivity analysis to examine the robustness of conclusions to different assumptions about departures from the stated protocol.

In addition to the cross-sectional analysis at 4 months, we will also perform a longitudinal analysis of EQ-5D data collected at all study time points (1, 4 and 12 months) to a single value, namely the area under the curve (AUC) and facilitated comparisons of the AUCs between treatment groups. It is advisable in the presence of missing data to use summary statistics generated by mixed models, as estimates will not be biased under the assumption that data are missing at random, or completely at random. Therefore we will fit a repeated measures mixed model, with the same fixed effect structure as used in the primary analyses, but with a three level random effects structure where observations (time points) are nested within participants, and participants nested within recruitment centre.

It seems likely that some data may not be available due to voluntary withdrawal of patients, lack of completion of individual data items or general loss to follow-up. Where possible the reasons for data 'missingness' will be ascertained and reported. Although missing data are not expected to be a problem for this study, the nature and pattern of the missingness will be carefully considered — including in particular whether data can be treated as missing at random (MAR). If judged appropriate, missing data will be imputed using the multiple imputation facilities available in R.[18] Any imputation methods used for scores and other derived variables will be carefully considered and justified. If the degree of missingness is relatively low, as

For peer review only

expected, the primary analysis will be based on complete cases only (complete case analysis), with analysis of imputed datasets used to assess the sensitivity of the analysis to the missing data. Reasons for ineligibility, non-compliance, withdrawal or other protocol violations will be stated and any patterns summarized. More formal analysis, for example using logistic regression with 'protocol violation' as a response, may also be appropriate and aid interpretation.

Secondary analyses will be undertaken using the strategy described for the primary outcome for other approximately normally distributed outcome measures; e.g. EQ-5D at 12 months. For dichotomous outcome variables, such as indicators of adverse events and other complications related to the trial interventions, mixed effects logistic regression analysis will be undertaken with results presented as odds ratios (and 95% confidence intervals) between the trial groups. The temporal patterns of any complications will be presented graphically and if appropriate a time-to-event analysis (Kaplan-Meier survival analysis) will be used to assess the overall risk and risk within individual classes of complications (e.g. revision) and death. Cox's proportional hazards regression will be used to test for differences in death rates between the trial intervention groups, after adjusting for age and gender. Multiple complications will be defined as two or more independent events, i.e. not continuations of a previous complication, for the same patient and will be identified only after discussion with the clinical team.

Economic analysis

A within-trial cost-effectiveness analysis of cemented versus uncemented hemiarthroplasty in participants over 60 years of age with a displaced intracapsular hip fracture will be conducted from the UK NHS and Personal Social Services perspective (PSS)[19] in the base case analysis. Resource utilisation involving differences in surgical treatments between the two intervention groups will be obtained from case report forms (CRFs) that would be completed by the local research teams. Broader resource utilisation will be captured through CRFs and patient questionnaires administered at baseline, 1 month, 4 months, and 12 months post-injury. Unit costs for health and social care resources will largely be derived from the latest available local and national sources and estimated in line with best practice. Costs will be standardised to current prices where appropriate. An incremental cost-effectiveness analysis, expressed in terms of incremental cost per quality-adjusted life year (QALY) gained, will be performed. Results will be presented using incremental cost-effectiveness ratios (ICERs), net monetary benefit, and cost effectiveness acceptability curves (CEACs) generated via non-parametric bootstrapping. Multiple imputation methods will be used to impute missing data and avoid biases associated with complete case analysis. Sensitivity analyses involving economic analysis from the societal perspective and extending the time frame from four months to one year will also be conducted.

Trial organisation and oversight

The day-to-day management of the trial will be the responsibility of the trial manager, based at the Nuffield Department of Orthopaedics, Rheumatology and

For peer review only - http://bmjopen.bmj.com/site/about/guidelines.xhtml

Musculoskeletal Sciences and supported by OCTRU staff. This will be overseen by a trial management group, who will meet monthly to assess progress. It will be the responsibility of the trial manager to undertake training of the research associates at each of the study centres. The study statistician and health economist will be closely involved in setting up data capture systems, design of databases and clinical reporting forms.

A Trial Steering Committee (TSC) and an independent DAMOCLES[20] compliant Data and Safety Monitoring Committee (DSMC), that will assess progress, conduct and participant safety, will be set up at the start of the study.

Quality control

Quality control procedures will be undertaken during recruitment and data collection phases of the study to ensure research is conducted, generated, recorded and reported in compliance with the protocol, GCP and ethics committee. The chief investigator and the trial manager will develop data management and monitoring plans.

Ethics and dissemination

The results of this trial will be disseminated to the hip fracture clinical community via presentations at national and international meetings as well as publication in peer-reviewed journals. Authorship will be determined in accordance with the International Committee of Medical Journal Editors (ICMJE) guidelines, and other contributors will be acknowledged. All data will be presented such that no individual participants can be identified. A lay summary informing patients and the public of the trial outcome will be available on the trial website. Further documentation suitable for the general patient and public communities will be prepared where appropriate by the research team in collaboration with lay representatives.

Contributors

MLC, JA, NP, MAF, MD, AM, JG and XG were responsible for obtaining grant funding for this trial. MAF, MLC, JA, RL, KM, MEP, AM, JG, NP and XG developed the trial protocol and contributed to writing the manuscript. NP developed the statistical analysis plan and is leading the statistical analysis for the study. MD and MEP developed the economic analysis plan and MEP is leading the economic analysis for the study. All authors reviewed and agreed the final manuscript.

Funding

This project is funded by the National Institute for Health Research (NIHR), Research for Patient Benefit (PB-PG-0215-36043 and PB-PG-1216-20021) and supported by the NIHR Oxford Biomedical Research Centre.

Ethics approval

Wales Research Ethics Committee 5 granted ethical approval for the feasibility phase on 2nd December 2016 (16/WA/0351) and the definitive trial on 22nd November 2017 (17/WA/0383).

Competing interests

For peer review only

MC is a National Institute for Health Research (NIHR) Senior Investigator. The views expressed in this article are those of the author(s) and not necessarily those of the NIHR, or the Department of Health and Social Care.
XG is funded by a National Institute for Health Research Clinician Scientist Grant. Further funding from industry and charitable grants are and have been made available to his institution. He has ongoing expert consultancy with several companies; none involve the development of any implant for use in hip fracture care. All decisions relating to the design, conduct, analysis, write-up and publication of research are independent of these funders. He has ongoing expert consultancy with several companies; none involve the development of any implant for use in hip fracture care.

Disclaimer
The views expressed are those of the authors.

REFERENCES

1 Falls and Fragility Fracture Audit Programme (FFFAP). Royal College of Physicians 2016.

2 No authors listed. Hip fracture: Management. *NICE* 2017.

3 Parker MJ, Gurusamy KS, Azegami S. Arthroplasties (with and without bone cement) for proximal femoral fractures in adults. *Cochrane Database Syst Rev* 2010;**6**:CD001706. doi:10.1002/14651858.CD001706.pub4

4 Veldman HD, Heyligers IC, Grimm B, *et al.* Cemented versus cementless hemiarthroplasty for a displaced fracture of the femoral neck: a systematic review and meta-analysis of current generation hip stems. *The Bone and Joint Journal* 2017;**99-B**:421–31. doi:10.1302/0301-620X.99B4.BJJ-2016-0758.R1

5 Griffin XL, Achten J, Parsons N, *et al.* The Warwick Hip Trauma Evaluation -an abridged protocol for the WHiTE Study: A multiple embedded randomised controlled trial cohort study. *Bone and Joint Research* 2012;**1**:310–4. doi:10.1302/2046-3758.111.2000127

6 *EuroQol*. https://euroqol.org/eq-5d-instruments/eq-5d-5l-about/valuation-standard-value-sets/crosswalk-index-value-calculator/ (accessed 27 Aug2019).

7 Herdman M, Gudex C, Lloyd A, *et al.* Development and preliminary testing of the new five-level version of EQ-5D (EQ-5D-5L). *Qual Life Res* 2011;**20**:1727–36. doi:10.1007/s11136-011-9903-x

8 van Hout B, Janssen MF, Feng Y-S, *et al.* Interim scoring for the EQ-5D-5L: mapping the EQ-5D-5L to EQ-5D-3L value sets. *Value Health* 2012;**15**:708–15. doi:10.1016/j.jval.2012.02.008

9 Haywood KL, Griffin XL, Achten J, *et al.* Developing a core outcome set for hip

For peer review only

fracture trials. *The Bone and Joint Journal* 2014;**96-B**:1016–23. doi:10.1302/0301-620X.96B8.33766

10 Parsons N, Griffin XL, Achten J, *et al.* Outcome assessment after hip fracture: is EQ-5D the answer? *Bone and Joint Research* 2014;:69–75. doi:10.1302/2046-3758.33.2000250

11 Tidermark JJ, Bergström GG. Responsiveness of the EuroQol (EQ-5D) and the Nottingham Health Profile (NHP) in elderly patients with femoral neck fractures. *Qual Life Res* 2007;**16**:321–30. doi:10.1007/s11136-006-9004-4

12 Bryan S, Hardyman W, Bentham P, *et al.* Proxy completion of EQ-5D in patients with dementia. *Qual Life Res* 2005;**14**:107–18. doi:10.2307/4038189

13 Hounsome N, Orrell M, Edwards RT. EQ-5D as a Quality of Life Measure in People with Dementia and Their Carers: Evidence and Key Issues. *Value Health* 2011;**14**:390–9. doi:10.1016/j.jval.2010.08.002

14 Walters SJ, Brazier JE. Comparison of the minimally important difference for two health state utility measures: EQ-5D and SF-6D. *Qual Life Res* 2005;**14**:1523–32. doi:10.2307/4039890

15 Cohen JJ. *Statistical power analysis for the behavioural sciences*. Erlbaum 1988.

16 Parsons N, Griffin XL, Achten J, *et al.* Modelling and estimation of health-related quality of life after hip fracture: A re-analysis of data from a prospective cohort study. *Bone and Joint Research* 2018;**7**:1–5. doi:10.1302/2046-3758.71.BJR-2017-0199

17 *R: A language and environment for statistical computing. R Foundation for Statistical Computing.* R Core Team (2018) https://www.R-project.org/ (accessed 3 Jul2019).

18 van Buuren S, Groothuis-Oudshoorn K. mice: Multivariate Imputation by Chained Equations in R. *Journal of Statistical Software* 2011;**45(3)**:1–67.https://www.jstatsoft.org/v45/i03/

19 NICE. Guide to methods of technology appraisal. April 4, 2013. Available from https://www.nice.org.uk/. [Accessed December 1, 2014].

20 DAMOCLES Study Group, NHS Health Technology Assessment Programme. A proposed charter for clinical trial data monitoring committees: helping them to do their job well. Lancet. 2005;**365**:711–22. doi:10.1016/S0140-6736(05)17965-3

For peer review only

STANDARD PROTOCOL ITEMS: RECOMMENDATIONS FOR INTERVENTIONAL TRIALS

SPIRIT 2013 Checklist: Recommended items to address in a clinical trial protocol and related documents*

Section/item	Item No	Description
**Administrative information**		
Title	1	Descriptive title identifying the study design, population, interventions, and, if applicable, trial acronym
Trial registration	2a	Trial identifier and registry name. If not yet registered, name of intended registry
	2b	All items from the World Health Organization Trial Registration Data Set
Protocol version	3	Date and version identifier
Funding	4	Sources and types of financial, material, and other support
Roles and responsibilities	5a	Names, affiliations, and roles of protocol contributors
	5b	Name and contact information for the trial sponsor
	5c	Role of study sponsor and funders, if any, in study design; collection, management, analysis, and interpretation of data; writing of the report; and the decision to submit the report for publication, including whether they will have ultimate authority over any of these activities
	5d	Composition, roles, and responsibilities of the coordinating centre, steering committee, endpoint adjudication committee, data management team, and other individuals or groups overseeing the trial, if applicable (see Item 21a for data monitoring committee)
**Introduction**		
Background and rationale	6a	Description of research question and justification for undertaking the trial, including summary of relevant studies (published and unpublished) examining benefits and harms for each intervention
	6b	Explanation for choice of comparators
Objectives	7	Specific objectives or hypotheses
Trial design	8	Description of trial design including type of trial (eg, parallel group, crossover, factorial, single group), allocation ratio, and framework (eg, superiority, equivalence, noninferiority, exploratory)

For peer review only

Methods: Participants, interventions, and outcomes

Study setting	9	Description of study settings (eg, community clinic, academic hospital) and list of countries where data will be collected. Reference to where list of study sites can be obtained
Eligibility criteria	10	Inclusion and exclusion criteria for participants. If applicable, eligibility criteria for study centres and individuals who will perform the interventions (eg, surgeons, psychotherapists)
Interventions	11a	Interventions for each group with sufficient detail to allow replication, including how and when they will be administered
	11b	Criteria for discontinuing or modifying allocated interventions for a given trial participant (eg, drug dose change in response to harms, participant request, or improving/worsening disease)
	11c	Strategies to improve adherence to intervention protocols, and any procedures for monitoring adherence (eg, drug tablet return, laboratory tests)
	11d	Relevant concomitant care and interventions that are permitted or prohibited during the trial
Outcomes	12	Primary, secondary, and other outcomes, including the specific measurement variable (eg, systolic blood pressure), analysis metric (eg, change from baseline, final value, time to event), method of aggregation (eg, median, proportion), and time point for each outcome. Explanation of the clinical relevance of chosen efficacy and harm outcomes is strongly recommended
Participant timeline	13	Time schedule of enrolment, interventions (including any run-ins and washouts), assessments, and visits for participants. A schematic diagram is highly recommended (see Figure)
Sample size	14	Estimated number of participants needed to achieve study objectives and how it was determined, including clinical and statistical assumptions supporting any sample size calculations
Recruitment	15	Strategies for achieving adequate participant enrolment to reach target sample size

Methods: Assignment of interventions (for controlled trials)

Allocation:

Sequence generation	16a	Method of generating the allocation sequence (eg, computer-generated random numbers), and list of any factors for stratification. To reduce predictability of a random sequence, details of any planned restriction (eg, blocking) should be provided in a separate document that is unavailable to those who enrol participants or assign interventions

For peer review only - http://bmjopen.bmj.com/site/about/guidelines.xhtml

Allocation concealment mechanism	16b	Mechanism of implementing the allocation sequence (eg, central telephone; sequentially numbered, opaque, sealed envelopes), describing any steps to conceal the sequence until interventions are assigned
Implementation	16c	Who will generate the allocation sequence, who will enrol participants, and who will assign participants to interventions
Blinding (masking)	17a	Who will be blinded after assignment to interventions (eg, trial participants, care providers, outcome assessors, data analysts), and how
	17b	If blinded, circumstances under which unblinding is permissible, and procedure for revealing a participant's allocated intervention during the trial
**Methods: Data collection, management, and analysis**		
Data collection methods	18a	Plans for assessment and collection of outcome, baseline, and other trial data, including any related processes to promote data quality (eg, duplicate measurements, training of assessors) and a description of study instruments (eg, questionnaires, laboratory tests) along with their reliability and validity, if known. Reference to where data collection forms can be found, if not in the protocol
	18b	Plans to promote participant retention and complete follow-up, including list of any outcome data to be collected for participants who discontinue or deviate from intervention protocols
Data management	19	Plans for data entry, coding, security, and storage, including any related processes to promote data quality (eg, double data entry; range checks for data values). Reference to where details of data management procedures can be found, if not in the protocol
Statistical methods	20a	Statistical methods for analysing primary and secondary outcomes. Reference to where other details of the statistical analysis plan can be found, if not in the protocol
	20b	Methods for any additional analyses (eg, subgroup and adjusted analyses)
	20c	Definition of analysis population relating to protocol non-adherence (eg, as randomised analysis), and any statistical methods to handle missing data (eg, multiple imputation)
**Methods: Monitoring**		
Data monitoring	21a	Composition of data monitoring committee (DMC); summary of its role and reporting structure; statement of whether it is independent from the sponsor and competing interests; and reference to where further details about its charter can be found, if not in the protocol. Alternatively, an explanation of why a DMC is not needed

	21b	Description of any interim analyses and stopping guidelines, including who will have access to these interim results and make the final decision to terminate the trial
Harms	22	Plans for collecting, assessing, reporting, and managing solicited and spontaneously reported adverse events and other unintended effects of trial interventions or trial conduct
Auditing	23	Frequency and procedures for auditing trial conduct, if any, and whether the process will be independent from investigators and the sponsor
**Ethics and dissemination**		
Research ethics approval	24	Plans for seeking research ethics committee/institutional review board (REC/IRB) approval
Protocol amendments	25	Plans for communicating important protocol modifications (eg, changes to eligibility criteria, outcomes, analyses) to relevant parties (eg, investigators, REC/IRBs, trial participants, trial registries, journals, regulators)
Consent or assent	26a	Who will obtain informed consent or assent from potential trial participants or authorised surrogates, and how (see Item 32)
	26b	Additional consent provisions for collection and use of participant data and biological specimens in ancillary studies, if applicable
Confidentiality	27	How personal information about potential and enrolled participants will be collected, shared, and maintained in order to protect confidentiality before, during, and after the trial
Declaration of interests	28	Financial and other competing interests for principal investigators for the overall trial and each study site
Access to data	29	Statement of who will have access to the final trial dataset, and disclosure of contractual agreements that limit such access for investigators
Ancillary and post-trial care	30	Provisions, if any, for ancillary and post-trial care, and for compensation to those who suffer harm from trial participation
Dissemination policy	31a	Plans for investigators and sponsor to communicate trial results to participants, healthcare professionals, the public, and other relevant groups (eg, via publication, reporting in results databases, or other data sharing arrangements), including any publication restrictions
	31b	Authorship eligibility guidelines and any intended use of professional writers
	31c	Plans, if any, for granting public access to the full protocol, participant-level dataset, and statistical code

For peer review only

Appendices

Informed consent materials	32	Model consent form and other related documentation given to participants and authorised surrogates
Biological specimens	33	Plans for collection, laboratory evaluation, and storage of biological specimens for genetic or molecular analysis in the current trial and for future use in ancillary studies, if applicable

*It is strongly recommended that this checklist be read in conjunction with the SPIRIT 2013 Explanation & Elaboration for important clarification on the items. Amendments to the protocol should be tracked and dated. The SPIRIT checklist is copyrighted by the SPIRIT Group under the Creative Commons "Attribution-NonCommercial-NoDerivs 3.0 Unported" license.

For peer review only

For peer review only - http://bmjopen.bmj.com/site/about/guidelines.xhtml

BMJ Open

Randomised controlled trial comparing hydroxyapatite coated uncemented hemiarthroplasty with cemented hemiarthroplasty for the treatment of displaced intracapsular hip fractures: a protocol for the WHITE 5 study

Journal:	*BMJ Open*
Manuscript ID	bmjopen-2019-033957.R1
Article Type:	Protocol
Date Submitted by the Author:	12-Nov-2019
Complete List of Authors:	Fernandez, Miguel; University of Oxford Nuffield Department of Orthopaedics Rheumatology and Musculoskeletal Sciences, Oxford Trauma; University Hospitals Coventry and Warwickshire NHS Trust, Trauma & Orthopaedic Surgery<br>Achten, Juul; University of Oxford, NDORMS<br>Lerner, Robin; University of Oxford Nuffield Department of Orthopaedics Rheumatology and Musculoskeletal Sciences, Oxford Trauma<br>Mironov, Katy; University of Oxford Nuffield Department of Orthopaedics Rheumatology and Musculoskeletal Sciences, Oxford Trauma<br>Parsons, Nicholas; University of Warwick, Warwick Medical School<br>Dritsaki, Melina; University of Warwick,<br>Png, May ee; University of Oxford, NDORMS<br>McGibbon, Alwin; Patient representative, UNTRAP University of Warwick<br>Gould, Jenny; Patient representative<br>Griffin, Xavier; University of Oxford Nuffield Department of Orthopaedics Rheumatology and Musculoskeletal Sciences, Kadoorie Centre<br>Costa, Matthew; University of Oxford, Nuffield Department of Orthopaedics, Rheumatology, and Musculoskeletal Sciences
**Primary Subject Heading**:	Surgery
Secondary Subject Heading:	Surgery
Keywords:	hip fracture, randomised controlled trial, hemiarthroplasty

SCHOLARONE™
Manuscripts

Randomised controlled trial comparing hydroxyapatite coated uncemented hemiarthroplasty with cemented hemiarthroplasty for the treatment of displaced intracapsular hip fractures: a protocol for the WHITE 5 study

M. A. Fernandez[1,2], J. Achten[1], R. G. Lerner[1], K. Mironov[1], N. Parsons[3], M. Dritsaki[4], M. E. Png[4], A. McGibbon[5], J. Gould[5], X. L. Griffin[1], and M. L. Costa[1*]

[1]Oxford Trauma, NDORMS, University of Oxford, Oxford OX3 9DU, UK

[2]University Hospitals Coventry & Warwickshire NHS Trust, Clifford Bridge Road, Coventry, CV2 2DX, UK

[3]Statistics and epidemiology unit, Warwick Medical School, University of Warwick, Gibbet Hill Road, Coventry, CV4 7AL, UK

[4]Oxford Clinical Trial Unit, NDORMS, University of Oxford, Oxford OX3 9DU, UK

[5]Patient representatives

*corresponding author: Prof Matthew Costa, Oxford Trauma, NDORMS, Kadoorie Centre, John Radcliffe Hospital, Headley Way, Oxford OX3 9DU, UK. Matthew.costa@ndorms.ox.ac.uk

Word count
4567

Key words
Hip fracture, hemiarthroplasty, randomised controlled trial

For peer review only

ABSTRACT

Introduction
Hip fracture is a serious injury in adults, especially those aged over 60 years. The most common type of hip fracture (displaced intracapsular) is treated for the majority of patients with a partial hip replacement (hemiarthroplasty). The hemiarthroplasty implant can be fixed to the bone with or without bone cement. Cement is the current recommended technique but recently some risks have been identified which could potentially be avoided by using uncemented implants. Controversy therefore remains about which type of hemiarthroplasty offers patients the best outcomes.
This is the protocol for a multicentre randomised controlled trial comparing cemented hemiarthroplasty versus uncemented hemiarthroplasty for patients 60 years and over with a displaced intracapsular hip fracture.

Methods and analysis
Multi-centre (a minimum of 7 UK hospitals), multi-surgeon, parallel group, two-arm, superiority, randomised controlled trial. Patients aged 60 years and older with a displaced intracapsular hip fracture treated with hemiarthroplasty surgery are eligible. Participants will be randomly allocated on a 1:1 basis to either a cemented hemiarthroplasty or a modern hydroxyapatite coated uncemented hemiarthroplasty. Otherwise all care will be in accordance with the National Institute for Health and Care Excellence guidance. A minimum of 1128 patients will be recruited to obtain 90% power to detect a 0.075-point difference in the primary endpoint: health-related quality of life (EuroQol 5 Dimensions 5 Levels EQ-5D-5L) at 4-months post-injury. The treatment effect will be estimated using a two-sided t-test adjusted for age, gender and cognitive impairment based on an intention-to-treat analysis. Secondary outcomes include mortality, complications including revision surgery and cause, mobility status, residential status, health-related quality of life at 1 and 12-months and health resource use. A within-trial economic analysis will be conducted.

Ethics, dissemination and funding
Wales Research Ethics Committee 5 approved the feasibility phase on 2nd December 2016 (16/WA/0351) and the definitive trial on 22nd November 2017 (17/WA/0383). This study is sponsored by the University of Oxford and funded by the National Institute for Health Research, Research for Patient Benefit (PB-PG-0215-36043 and PB-PG-1216-20021). A manuscript for a peer-reviewed journal will be prepared and the results shared with patients via local mechanisms at participating centres.

Trial Registration number: ISRCTN18393176

Strengths & Limitations
- Pragmatic multicentre randomised controlled trial
- Powered to detect differences in health-related quality of life
- Inclusion of participants with and without cognitive impairment
- Outcomes include the UK core outcome set for hip fracture

For peer review only

- The trial will not capture late complications, implant failure or revision surgery beyond 1-year post injury

INTRODUCTION

Hip fracture is one of the biggest challenges facing patients and healthcare systems. Every year in the UK there are more than 65,000 hip fractures.[1] Displaced 'intracapsular' fractures represent approximately half of all hip fractures. In these fractures, the head of the femur is broken off at the neck. The blood supply to the femoral head is tenuous and, even if the fracture is fixed back in to its anatomical position, fracture healing is unreliable and 40% of patients will have a failure of fixation. For this reason, arthroplasty (replacement) of the femoral head is preferred in the UK. A minority of patients receive a 'total hip arthroplasty' (replacing the 'socket' of the hip joint as well as the femoral head) if they are particularly active before the fracture, but in most cases only the head of the femur is replaced; a hemiarthroplasty.

The National Institute for Health and Care Excellence (NICE)[2] guidelines for hip fracture recommend the use of bone cement to aid in the fixation of the hemiarthroplasty implant to the bone. These guidelines are based on outcomes of studies which show less postoperative pain and better function when cemented components were used compared to their first generation uncemented counterparts (e.g. the Austin Moore prosthesis). The evidence base for these guidelines are summarised in a Cochrane review[3] which acknowledged the lack of evidence for more contemporary uncemented implants and concluded that "there is still a place for trials of contemporary uncemented stems with cemented stem hemiarthroplasty". A recent independent systematic review and meta-analysis compared cemented hemiarthroplasty with modern uncemented hemiarthroplasty and concluded: *"there remains a need for a methodologically sound, large muticentre RCT comparing modern cemented and cementless hemiarthroplasty stems in the medium – and long-term, not only focusing on mortality and complications but also on patient reported outcome measures."*[4]

This is the protocol for a multi-centre parallel-group randomised controlled trial, that compares cemented hemiarthroplasty and modern hydroxyapatite coated uncemented hemiarthroplasty.

AIMS AND OBJECTIVES

The aim of this randomised controlled trial is to compare health-related quality of life (HRQoL) in participants over 60 years of age with a displaced intracapsular hip fracture receiving a modern hydroxyapatite coated uncemented hemiarthroplasty versus the current standard-of-care cemented hemiarthroplasty.

The primary objective is:

- To quantify and draw inferences on observed differences in participants' HRQoL between the trial treatment groups at 4 months post-injury.

The secondary objectives are:

For peer review only - http://bmjopen.bmj.com/site/about/guidelines.xhtml

- To quantify and draw inferences on the observed differences in participants' HRQoL between the trial treatment groups at 1 and 12 months post-injury
- To quantify and draw inferences on the observed differences in the proportion of complications, including further surgery, within the first 12 months post-injury between the trial treatment groups
- To quantify and draw inferences on observed differences in mortality, mobility and residential status at 1, 4 and 12 months post-injury
- To investigate, using appropriate statistical and economic analytical methods, the resource use, costs and incremental cost effectiveness of the trial treatment groups at four months post-injury

METHODS AND ANALYSIS

Study design

A multi-centre, parallel group, two-arm, superiority, standard-of-care randomised controlled superiority trial in a minimum of 7 UK recruitment centres. Each of these recruitment centres is recruiting hip fracture patients as part of the World Hip Trauma Evaluation (WHiTE) hip fracture cohort study.[5] The study is conducted in two phases: an initial feasibility phase in which the acceptability of the interventions and trial processes were tested, and a definitive phase which comprises the main trial. Data from the feasibility phase will be locked, and not analysed, at completion. At the end of the definitive main trial phase, data from the two phases will be analysed together as a single dataset.

Trial Registration

Application for trial registration was made to the ISRCTN on 7th March 2017 prior to commencing patient enrolment.

Patient and Public Involvement

At the centre of this work is the potential for patient benefit by reducing the risks of hip fracture surgery and improving patient outcomes. The involvement of patient and public representatives has therefore been a key element of this work to ensure that the running of the trial remains patient focused. JG and AM are patient representative coauthors who have been involved in all aspects of this work from designing the trial, obtaining grant funding, developing the trial materials, and participation in the running of the trial. More specifically, the role of patient representatives has been to ensure that trial processes and information are focused on patients, to provide the patient perspective as members of the trial management group, and to ensure that all trial processes have taken into account the needs of patients both with and without cognitive impairment.

Eligibility

Patients will be screened against the following criteria:

Inclusion criteria

- All patients, both those with and without capacity, presenting with a displaced intracapsular fracture of the hip suitable for hemiarthroplasty.

For peer review only

Exclusion criteria

- Patients younger than 60 years of age
- Patients who are managed non-operatively
- Patients who are treated with a total hip replacement

Consent

Patients with a hip fracture are a clinical priority for urgent operative care. They undergo surgery on the next available trauma operating list. All patients with a fracture of the hip are in pain and will have received opiate analgesia. It is therefore understandable that the majority of patients find the initial period of their treatment in hospital confusing and disorientating. Similarly, patients' next of kin, carers and friends are often anxious at this time and may have difficulty in absorbing the large amounts of information that they are given about the injury and plan for treatment. In this emergency situation the focus is on obtaining consent for surgery (where possible) and on informing the patient and any next of kin about immediate clinical care. It is often not possible for the patient, relative or carer (consultee) to review trial documentation, consider the information and communicate an informed decision about whether they would wish to participate in the study. The consent procedure for this trial will reflect that of the surgery, with the clinical team assessing capacity before taking consent for the surgical procedure, and this capacity assessment then being used to guide the proper approach to consenting to the research. An appropriate method, in line with the mental capacity act and approved by the National Research Ethics Committee, will be used to gain either prospective or retrospective consent from the patient or appropriate consultee by a Good Clinical Practice (GCP)-trained, appropriately delegated member of the research team.

Randomisation and blinding

The allocation sequence will be computer generated by the trial statistician and administered, using secure online randomisation, by Oxford Clinical Trials Research Unit (OCTRU), University of Oxford. Participants will be enrolled by the operating surgeon or trial research associate. The research associate will receive notification of the allocated treatment and inform the surgeon and the operating theatre staff. The randomisation algorithm will use variable block sizes (2, 4 and 6), to ensure good treatment balance, at each of the recruitment centres. The surgery will be performed under the care of the consultant surgeon on-call at the time the patient is admitted as per local practice in the recruitment centres. The large number of surgeons – previous experience in similar trials suggests over 200 surgeons will take part – and the wide skill mix should minimise any surgeon-specific effects such that stratification by surgeon is unnecessary. In order to negate bias in the self-reported HRQoL outcome measures participants will be blinded to treatment allocation. The operating surgeon cannot be blinded to the allocation but they will not be involved in the assessment of outcomes. Patients will be blinded until the completion of the trial when the blinding will be broken if requested by the patients. There will be no formal analysis of the success of the blinding.

For peer review only

Post-randomisation withdrawals and exclusion

Throughout the study, screening logs will be kept to determine the number of patients assessed for eligibility and reasons for any exclusion. Participants may withdraw from the study at any time without prejudice. If a participant withdraws from the study completely, data collected up until the point of withdrawal will be included in the final analysis. Patients who decline to continue to take part will be given the opportunity to discuss and inform the research team of the reasoning behind their decision not to take part. Similarly, data obtained from participants who die before they or a personal consultee has been approached about continued consent/agreement will be included in the final analysis.

Treatments

Standardised treatment pathway

Participants will usually be assessed in the Emergency Department. Diagnosis of a hip fracture will be confirmed by a plain radiograph, as per routine clinical care. Supplementary imaging will be at the discretion of the treating clinical team. Routine investigations, anaesthetic assessment, antibiotic and venous thromboembolic prophylaxis will be used as per local policy. A regional or general anaesthesia technique will be used for every participant as per routine clinical care. All participants will receive perioperative prophylactic antibiotics in accordance with current protocols agreed at each recruitment centre. Postoperative analgesia will be prescribed intra-operatively and reviewed by the responsible clinical teams as appropriate.

In the postoperative period, as per standard of care, all participants will undergo a physiotherapy and occupational therapy assessment to create a rehabilitation plan. The aim of this plan will be for participants to mobilise through early, active, full weight bearing. Participants will be discharged from the acute Orthopaedic Trauma Ward at the earliest safe opportunity to the most appropriate discharge destination as determined by the multi-disciplinary clinical team. Participants will be given the relevant NHS Trust information packs.

Interventions

Appropriate preparation, positioning and surgical technique will be left to the discretion of the operating surgeon, as per their normal clinical practice. Participants will be randomly allocated to one of two treatment groups:

Group 1: Cemented hemiarthroplasty. Replacement of the femoral head and neck with a cemented femoral stem and head as per NICE guidance; the current standard-of-care (control) intervention.

Group 2: Modern uncemented hemiarthroplasty. Replacement of the femoral head and neck with a modern (hydroxyapatite coated) uncemented hemiarthroplasty; the alternative intervention.

Both types of hip replacement are used routinely throughout the NHS. While the principles of both cemented and uncemented arthroplasty are inherent in the

For peer review only

technique, the surgical approach and other technical aspects of the surgery will be left to the discretion of the senior operating surgeon as per their normal practice. This will ensure that the results of this pragmatic trial can be extrapolated broadly across the NHS.

Outcomes and Data Management

Personal data collected during the study will be handled and stored in accordance with the 1998 Data Protection Act, which requires data to be anonymised as soon as it is practical to do so. The data collected from participants will be entered in linked-anonymised form to the trial database. All electronic patient-identifiable information will be stored on a secure, password-protected database at the University of Oxford, accessible only to the research team. Any paper copies of identifiable data, and corresponding re-identifying links to the participant trial ID, will be stored separately, in a locked cabinet in an access-restricted part of the Kadoorie Centre, John Radcliffe Hospital, Oxford. The study databases will be developed by the trial programmer and all specifications agreed between the trial programmer, statistician and trial manager and other relevant members of the trial team. The procedure for data entry will be documented in the data management plan. As per routine clinical care, the existing National Hip Fracture Database dataset will be collected via telephone interview or postal questionnaire. We will send data collected at follow up to the NHFD via a secure email account for them to upload using the participant's date of birth and NHS number as identifiers.

Primary outcome Measure

The study primary outcome measure is EuroQol 5 Dimensions 5 Levels (EQ-5D-5L) index score at 4-months post-injury. The index score is derived from the 5 health state domains using the crosswalk value data sets.[6] EQ-5D-5L is a validated, self-rated instrument comprising a visual analogue scale (VAS) measuring health and a health status instrument, consisting of a five-level response (no problems, some problems, moderate problems, severe problems and unable) for five domains related to daily activities[7]; (i) mobility, (ii) self-care, (iii) usual activities, (iv) pain and discomfort and (v) anxiety and depression. The EQ-5D-5L instrument [7] facilitates the generation of a utility score from a person's health related quality of life, which refers to the preference that individuals have for any particular set of health outcomes. As per the current NICE position statement, the responses to the EQ-5D-5L will be converted into multi-attribute utility scores using an approved "cross-walk" to the 3L instrument and its established time trade-off utility algorithm for the UK population.[8]. A respondent's EQ-VAS gives self-rated health on a scale where the endpoints are labelled 'best imaginable health state' (100) and 'worst imaginable health state' (0). EQ-5D is part of the UK Core outcome set[9] and has been shown to be responsive to change,[10,11] including when reported by proxy for those with cognitive impairment.[12,13]

Secondary outcome measures

HRQoL (*EQ-5D-5L)* at 1 and 12 months post-injury. Mortality, revision surgery and cause, and all complications will be obtained from the patients' medical record. Patient interviews and questionnaires will be used to assess mobility status (walking

For peer review only

ability indoors and outdoors) and residential status. An assessment of resource use will inform the economic analysis plan below.

Sample size

The best available evidence we have from data collected during previously performed studies in this patient population suggests that the standard deviation for EQ-5D-5L at 4 months post-injury is approximately 0.3 points.[10] The best available evidence for what constitutes a minimal important difference (MID) for EQ-5D comes from a review of MID estimates.[14] A review of the literature shows an estimated median value of 0.08 for the MID for EQ-5D. Using our previously established standard deviation (SD=0.33), this suggests a standardised effect size of approximately 0.24; a 'small to moderate effect' based on Cohen's criteria.[15] Assuming that the EQ-5D at 4 months post-injury has an approximate normal distribution, which Parsons et al[10] suggests is reasonable, and a 1:1 allocation ratio, then if the true difference between the experimental and control group EQ-5D means is in the range 0.07 to 0.08, we will need to recruit the below number of participants (see Table 1) in each group to be able to reject the null hypothesis that the population means are equal with probability (power) 0.8 and 0.9 and type I error rate of 5% (significance).

Taking the intermediate MCID of 0.075, for 80% (90%) power, we would need to recruit 253 (338) patients in both the experimental arm and in the control arm, 506 (676) in total. In this population we expect considerable loss to follow-up due mainly to patients declining consent to further follow-up and incapacity, so we have assumed that only 60% of recruited study participants will be available at the definitive endpoint at 4 months. This gives a total sample size of 844 for 80% power and 1128 for 90% power. Conservatively, we aim to recruit 1128 to ensure 90% power based upon these assumptions.

Table 1: Group sizes required following sample size calculations.

**Power**	**MCID**		
	**0.07**	**0.075**	**0.08**
**80%**	290	253	222
**90%**	387	338	297

Statistical analysis

The main analysis will investigate differences in the primary outcome measure, EQ-5D (Health Related Quality of Life; HRQoL), between the two treatment groups on an intention-to-treat (ITT) basis at 4 months post-injury on a complete case basis. The 'death-adjusted' ED-5D measure, imputing death as zero, is the preferred analysis option in this population, and will be used for all analyses.[16] An initial analysis will test for differences between treatment groups using linear regression analysis, based on a Normal approximation for EQ-5D. Tests will be two-sided and considered to provide evidence for a significant difference if p-values are less than 0.05 (5% significance level). Estimates of treatment effects will be presented with 95% confidence intervals.

For peer review only

The stratification used for randomization will ensure balance in treatment allocation across recruitment centres. In addition to the unadjusted analyses we will also undertake regression analyses to adjust for any imbalance between treatment groups in patient baseline (pre-injury) EQ-5D, age and gender. Age and gender are likely to be reported by all study participants, however if baseline EQ-5D is poorly reported, such that it reduces the available sample size for the definitive analysis, then it will not be included in the definitive model. The fixed effects analysis (linear regression model) will be generalized by adding a random effect for recruitment centre to allow for possible heterogeneity in patient outcomes due more generally to the recruitment centre. The adjusted mixed-effects regression will be the definitive (primary) analyses and will be reported as such. Analyses will be undertaken using the specialist mixed-effects modelling functions available in the software package R (http://www.r-project.org/).[17] EQ-5D data will be assumed to be approximately normally distributed; possibly after appropriate variance-stabilising transformation. The primary focus will be the comparison of the two treatment groups of patients, and this will be reflected in the analysis which will be reported together with appropriate diagnostic plots that check the underlying model assumptions. Results will be presented as mean differences between the trial groups, with 95% confidence intervals. The primary analysis will be on an ITT basis. However, a per-protocol (PP) analysis will also be undertaken by repeating the primary outcome analysis using the actual treatment received by participants (in contrast to the allocated treatment) as a sensitivity analysis to examine the robustness of conclusions to different assumptions about departures from the stated protocol.

In addition to the cross-sectional analysis at 4 months, we will also perform a longitudinal analysis of EQ-5D data collected at all study time points (1, 4 and 12 months) to a single value, namely the area under the curve (AUC) and facilitated comparisons of the AUCs between treatment groups. It is advisable in the presence of missing data to use summary statistics generated by mixed models, as estimates will not be biased under the assumption that data are missing at random, or completely at random. Therefore we will fit a repeated measures mixed model, with the same fixed effect structure as used in the primary analyses, but with a three level random effects structure where observations (time points) are nested within participants, and participants nested within recruitment centre.

It seems likely that some data may not be available due to voluntary withdrawal of patients, lack of completion of individual data items or general loss to follow-up. Where possible the reasons for data 'missingness' will be ascertained and reported. Although missing data are not expected to be a problem for this study, the nature and pattern of the missingness will be carefully considered — including in particular whether data can be treated as missing at random (MAR). If judged appropriate, missing data will be imputed using the multiple imputation facilities available in R.[18] Any imputation methods used for scores and other derived variables will be carefully considered and justified. If the degree of missingness is relatively low, as

For peer review only

expected, the primary analysis will be based on complete cases only (complete case analysis), with analysis of imputed datasets used to assess the sensitivity of the analysis to the missing data. Reasons for ineligibility, non-compliance, withdrawal or other protocol violations will be stated and any patterns summarized. More formal analysis, for example using logistic regression with 'protocol violation' as a response, may also be appropriate and aid interpretation.

Secondary analyses will be undertaken using the strategy described for the primary outcome for other approximately normally distributed outcome measures; e.g. EQ-5D at 12 months. For dichotomous outcome variables, such as indicators of adverse events and other complications related to the trial interventions, mixed effects logistic regression analysis will be undertaken with results presented as odds ratios (and 95% confidence intervals) between the trial groups. The temporal patterns of any complications will be presented graphically and if appropriate a time-to-event analysis (Kaplan-Meier survival analysis) will be used to assess the overall risk and risk within individual classes of complications (e.g. revision) and death. Cox's proportional hazards regression will be used to test for differences in death rates between the trial intervention groups, after adjusting for age and gender. Multiple complications will be defined as two or more independent events, i.e. not continuations of a previous complication, for the same patient and will be identified only after discussion with the clinical team.

Economic analysis

A within-trial cost-effectiveness analysis of cemented versus uncemented hemiarthroplasty in participants over 60 years of age with a displaced intracapsular hip fracture will be conducted from the UK NHS and Personal Social Services perspective (PSS)[19] in the base case analysis. Resource utilisation involving differences in surgical treatments between the two intervention groups will be obtained from case report forms (CRFs) that would be completed by the local research teams. Broader resource utilisation will be captured through CRFs and patient questionnaires administered at baseline, 1 month, 4 months, and 12 months post-injury. Unit costs for health and social care resources will largely be derived from the latest available local and national sources and estimated in line with best practice. Costs will be standardised to current prices where appropriate. An incremental cost-effectiveness analysis, expressed in terms of incremental cost per quality-adjusted life year (QALY) gained, will be performed. Results will be presented using incremental cost-effectiveness ratios (ICERs), net monetary benefit, and cost effectiveness acceptability curves (CEACs) generated via non-parametric bootstrapping. Multiple imputation methods will be used to impute missing data and avoid biases associated with complete case analysis. Sensitivity analyses involving economic analysis from the societal perspective and extending the time frame from four months to one year will also be conducted.

Trial organisation and oversight

The day-to-day management of the trial will be the responsibility of the trial manager, based at the Nuffield Department of Orthopaedics, Rheumatology and

For peer review only

Musculoskeletal Sciences and supported by OCTRU staff. This will be overseen by a trial management group, who will meet monthly to assess progress. It will be the responsibility of the trial manager to undertake training of the research associates at each of the study centres. The study statistician and health economist will be closely involved in setting up data capture systems, design of databases and clinical reporting forms.

A Trial Steering Committee (TSC) and an independent DAMOCLES[20] compliant Data and Safety Monitoring Committee (DSMC), that will assess progress, conduct and participant safety, will be set up at the start of the study.

Quality control

Quality control procedures will be undertaken during recruitment and data collection phases of the study to ensure research is conducted, generated, recorded and reported in compliance with the protocol, GCP and ethics committee. The chief investigator and the trial manager will develop data management and monitoring plans.

Ethics and dissemination

Wales Research Ethics Committee 5 approved the feasibility phase on 2nd December 2016 (16/WA/0351) and the definitive trial on 22nd November 2017 (17/WA/0383). The results of this trial will be disseminated to the hip fracture clinical community via presentations at national and international meetings as well as publication in peer-reviewed journals. Authorship will be determined in accordance with the International Committee of Medical Journal Editors (ICMJE) guidelines, and other contributors will be acknowledged. All data will be presented such that no individual participants can be identified. A lay summary informing patients and the public of the trial outcome will be available on the trial website. Further documentation suitable for the general patient and public communities will be prepared where appropriate by the research team in collaboration with lay representatives.

Contributors

MLC, JA, NP, MAF, MD, AM, JG and XG were responsible for obtaining grant funding for this trial. MAF, MLC, JA, RL, KM, MEP, AM, JG, NP and XG developed the trial protocol and contributed to writing the manuscript. NP developed the statistical analysis plan and is leading the statistical analysis for the study. MD and MEP developed the economic analysis plan and MEP is leading the economic analysis for the study. All authors reviewed and agreed the final manuscript.

Funding

This project is funded by the National Institute for Health Research (NIHR), Research for Patient Benefit (PB-PG-0215-36043 and PB-PG-1216-20021) and supported by the NIHR Oxford Biomedical Research Centre.

Ethics approval

Wales Research Ethics Committee 5 granted ethical approval for the feasibility phase on 2nd December 2016 (16/WA/0351) and the definitive trial on 22nd November 2017 (17/WA/0383).

For peer review only

Competing interests

MC is a National Institute for Health Research (NIHR) Senior Investigator. The views expressed in this article are those of the author(s) and not necessarily those of the NIHR, or the Department of Health and Social Care.
XG is funded by a National Institute for Health Research Clinician Scientist Grant. Further funding from industry and charitable grants are and have been made available to his institution. He has ongoing expert consultancy with several companies; none involve the development of any implant for use in hip fracture care. All decisions relating to the design, conduct, analysis, write-up and publication of research are independent of these funders. He has ongoing expert consultancy with several companies; none involve the development of any implant for use in hip fracture care.

Disclaimer

The views expressed are those of the authors.

REFERENCES

1 Falls and Fragility Fracture Audit Programme (FFFAP). Royal College of Physicians 2016.

2 No authors listed. Hip fracture: Management. *NICE* 2017.

3 Parker MJ, Gurusamy KS, Azegami S. Arthroplasties (with and without bone cement) for proximal femoral fractures in adults. *Cochrane Database Syst Rev* 2010;**6**:CD001706. doi:10.1002/14651858.CD001706.pub4

4 Veldman HD, Heyligers IC, Grimm B, *et al.* Cemented versus cementless hemiarthroplasty for a displaced fracture of the femoral neck: a systematic review and meta-analysis of current generation hip stems. *The Bone and Joint Journal* 2017;**99-B**:421–31. doi:10.1302/0301-620X.99B4.BJJ-2016-0758.R1

5 Griffin XL, Achten J, Parsons N, *et al.* The Warwick Hip Trauma Evaluation -an abridged protocol for the WHiTE Study: A multiple embedded randomised controlled trial cohort study. *Bone and Joint Research* 2012;**1**:310–4. doi:10.1302/2046-3758.111.2000127

6 *EuroQol*. https://euroqol.org/eq-5d-instruments/eq-5d-5l-about/valuation-standard-value-sets/crosswalk-index-value-calculator/ (accessed 27 Aug2019).

7 Herdman M, Gudex C, Lloyd A, *et al.* Development and preliminary testing of the new five-level version of EQ-5D (EQ-5D-5L). *Qual Life Res* 2011;**20**:1727–36. doi:10.1007/s11136-011-9903-x

8 van Hout B, Janssen MF, Feng Y-S, *et al.* Interim scoring for the EQ-5D-5L: mapping the EQ-5D-5L to EQ-5D-3L value sets. *Value Health* 2012;**15**:708–15.

For peer review only

doi:10.1016/j.jval.2012.02.008

9 Haywood KL, Griffin XL, Achten J, *et al.* Developing a core outcome set for hip fracture trials. *The Bone and Joint Journal* 2014;**96-B**:1016–23. doi:10.1302/0301-620X.96B8.33766

10 Parsons N, Griffin XL, Achten J, *et al.* Outcome assessment after hip fracture: is EQ-5D the answer? *Bone and Joint Research* 2014;:69–75. doi:10.1302/2046-3758.33.2000250

11 Tidermark JJ, Bergström GG. Responsiveness of the EuroQol (EQ-5D) and the Nottingham Health Profile (NHP) in elderly patients with femoral neck fractures. *Qual Life Res* 2007;**16**:321–30. doi:10.1007/s11136-006-9004-4

12 Bryan S, Hardyman W, Bentham P, *et al.* Proxy completion of EQ-5D in patients with dementia. *Qual Life Res* 2005;**14**:107–18. doi:10.2307/4038189

13 Hounsome N, Orrell M, Edwards RT. EQ-5D as a Quality of Life Measure in People with Dementia and Their Carers: Evidence and Key Issues. *Value Health* 2011;**14**:390–9. doi:10.1016/j.jval.2010.08.002

14 Walters SJ, Brazier JE. Comparison of the minimally important difference for two health state utility measures: EQ-5D and SF-6D. *Qual Life Res* 2005;**14**:1523–32. doi:10.2307/4039890

15 Cohen JJ. *Statistical power analysis for the behavioural sciences*. Erlbaum 1988.

16 Parsons N, Griffin XL, Achten J, *et al.* Modelling and estimation of health-related quality of life after hip fracture: A re-analysis of data from a prospective cohort study. *Bone and Joint Research* 2018;**7**:1–5. doi:10.1302/2046-3758.71.BJR-2017-0199

17 *R: A language and environment for statistical computing. R Foundation for Statistical Computing.* R Core Team (2018) https://www.R-project.org/ (accessed 3 Jul2019).

18 van Buuren S, Groothuis-Oudshoorn K. mice: Multivariate Imputation by Chained Equations in R. *Journal of Statistical Software* 2011;**45(3)**:1–67.https://www.jstatsoft.org/v45/i03/

19 NICE. Guide to methods of technology appraisal. April 4, 2013. Available from https://www.nice.org.uk/. [Accessed December 1, 2014].

20 DAMOCLES Study Group, NHS Health Technology Assessment Programme. A proposed charter for clinical trial data monitoring committees: helping them to do their job well. Lancet. 2005;**365**:711–22. doi:10.1016/S0140-6736(05)17965-3

For peer review only

SPIRIT

STANDARD PROTOCOL ITEMS: RECOMMENDATIONS FOR INTERVENTIONAL TRIALS

SPIRIT 2013 Checklist: Recommended items to address in a clinical trial protocol and related documents*

Section/item	Item No	Description
**Administrative information**		
Title	1	Descriptive title identifying the study design, population, interventions, and, if applicable, trial acronym – **title page**
Trial registration	2a	Trial identifier and registry name. If not yet registered, name of intended registry – **abstract**
	2b	All items from the World Health Organization Trial Registration Data Set – **N/A**
Protocol version	3	Date and version identifier – N/A
Funding	4	Sources and types of financial, material, and other support - **Abstract**
Roles and responsibilities	5a	Names, affiliations, and roles of protocol contributors – **title page**
	5b	Name and contact information for the trial sponsor – **abstract/title page**
	5c	Role of study sponsor and funders, if any, in study design; collection, management, analysis, and interpretation of data; writing of the report; and the decision to submit the report for publication, including whether they will have ultimate authority over any of these activities – **N/A**
	5d	Composition, roles, and responsibilities of the coordinating centre, steering committee, endpoint adjudication committee, data management team, and other individuals or groups overseeing the trial, if applicable (see Item 21a for data monitoring committee) – **pages 10-11 'Trial organisation and oversight'**
**Introduction**		
Background and rationale	6a	Description of research question and justification for undertaking the trial, including summary of relevant studies (published and unpublished) examining benefits and harms for each intervention – **page 3**
	6b	Explanation for choice of comparators – **page 3**
Objectives	7	Specific objectives or hypotheses – **page 3-4**

For peer review only

Trial design	8	Description of trial design including type of trial (eg, parallel group, crossover, factorial, single group), allocation ratio, and framework (eg, superiority, equivalence, noninferiority, exploratory) – **page 4 'Study design'**

Methods: Participants, interventions, and outcomes

Study setting	9	Description of study settings (eg, community clinic, academic hospital) and list of countries where data will be collected. Reference to where list of study sites can be obtained – **page 4 'Study Design'**
Eligibility criteria	10	Inclusion and exclusion criteria for participants. If applicable, eligibility criteria for study centres and individuals who will perform the interventions (eg, surgeons, psychotherapists) **- Page 4-5 'Eligibility'**
Interventions	11a	Interventions for each group with sufficient detail to allow replication, including how and when they will be administered – **page 6**
	11b	Criteria for discontinuing or modifying allocated interventions for a given trial participant (eg, drug dose change in response to harms, participant request, or improving/worsening disease) – **page 6**
	11c	Strategies to improve adherence to intervention protocols, and any procedures for monitoring adherence (eg, drug tablet return, laboratory tests) – **N/A**
	11d	Relevant concomitant care and interventions that are permitted or prohibited during the trial – **N/A**
Outcomes	12	Primary, secondary, and other outcomes, including the specific measurement variable (eg, systolic blood pressure), analysis metric (eg, change from baseline, final value, time to event), method of aggregation (eg, median, proportion), and time point for each outcome. Explanation of the clinical relevance of chosen efficacy and harm outcomes is strongly recommended – **page 3-4**
Participant timeline	13	Time schedule of enrolment, interventions (including any run-ins and washouts), assessments, and visits for participants. A schematic diagram is highly recommended (see Figure) – **N/A**
Sample size	14	Estimated number of participants needed to achieve study objectives and how it was determined, including clinical and statistical assumptions supporting any sample size calculations – **page 8**
Recruitment	15	Strategies for achieving adequate participant enrolment to reach target sample size – page 6

Methods: Assignment of interventions (for controlled trials)

Allocation:

For peer review only - http://bmjopen.bmj.com/site/about/guidelines.xhtml

Sequence generation	16a	Method of generating the allocation sequence (eg, computer-generated random numbers), and list of any factors for stratification. To reduce predictability of a random sequence, details of any planned restriction (eg, blocking) should be provided in a separate document that is unavailable to those who enrol participants or assign interventions – **page 5 in Randomisation and blinding**
Allocation concealment mechanism	16b	Mechanism of implementing the allocation sequence (eg, central telephone; sequentially numbered, opaque, sealed envelopes), describing any steps to conceal the sequence until interventions are assigned – **page 5**
Implementation	16c	Who will generate the allocation sequence, who will enrol participants, and who will assign participants to interventions – **page 5**
Blinding (masking)	17a	Who will be blinded after assignment to interventions (eg, trial participants, care providers, outcome assessors, data analysts), and how – **page 5**
	17b	If blinded, circumstances under which unblinding is permissible, and procedure for revealing a participant's allocated intervention during the trial – **page 5**
**Methods: Data collection, management, and analysis**		
Data collection methods	18a	Plans for assessment and collection of outcome, baseline, and other trial data, including any related processes to promote data quality (eg, duplicate measurements, training of assessors) and a description of study instruments (eg, questionnaires, laboratory tests) along with their reliability and validity, if known. Reference to where data collection forms can be found, if not in the protocol – **page 7 - 8**
	18b	Plans to promote participant retention and complete follow-up, including list of any outcome data to be collected for participants who discontinue or deviate from intervention protocols - **page 7 - 8**
Data management	19	Plans for data entry, coding, security, and storage, including any related processes to promote data quality (eg, double data entry; range checks for data values). Reference to where details of data management procedures can be found, if not in the protocol - **page 7**
Statistical methods	20a	Statistical methods for analysing primary and secondary outcomes. Reference to where other details of the statistical analysis plan can be found, if not in the protocol – **page 8-9**
	20b	Methods for any additional analyses (eg, subgroup and adjusted analyses) – **pages 8-10**
	20c	Definition of analysis population relating to protocol non-adherence (eg, as randomised analysis), and any statistical methods to handle missing data (eg, multiple imputation) – **pages 8-10**

Methods: Monitoring

Data monitoring	21a	Composition of data monitoring committee (DMC); summary of its role and reporting structure; statement of whether it is independent from the sponsor and competing interests; and reference to where further details about its charter can be found, if not in the protocol. – **Page 10-11**<br>Alternatively, an explanation of why a DMC is not needed
	21b	Description of any interim analyses and stopping guidelines, including who will have access to these interim results and make the final decision to terminate the trial – **page 11**
Harms	22	Plans for collecting, assessing, reporting, and managing solicited and spontaneously reported adverse events and other unintended effects of trial interventions or trial conduct – **page 11**
Auditing	23	Frequency and procedures for auditing trial conduct, if any, and whether the process will be independent from investigators and the sponsor – **N/A**

Ethics and dissemination

Research ethics approval	24	Plans for seeking research ethics committee/institutional review board (REC/IRB) approval – **abstract and page 11**
Protocol amendments	25	Plans for communicating important protocol modifications (eg, changes to eligibility criteria, outcomes, analyses) to relevant parties (eg, investigators, REC/IRBs, trial participants, trial registries, journals, regulators) – **page 11**
Consent or assent	26a	Who will obtain informed consent or assent from potential trial participants or authorised surrogates, and how (see Item 32) – **page 5**
	26b	Additional consent provisions for collection and use of participant data and biological specimens in ancillary studies, if applicable – **N/A**
Confidentiality	27	How personal information about potential and enrolled participants will be collected, shared, and maintained in order to protect confidentiality before, during, and after the trial – **page 7**
Declaration of interests	28	Financial and other competing interests for principal investigators for the overall trial and each study site – **page 12**
Access to data	29	Statement of who will have access to the final trial dataset, and disclosure of contractual agreements that limit such access for investigators – **page 11**
Ancillary and post-trial care	30	Provisions, if any, for ancillary and post-trial care, and for compensation to those who suffer harm from trial participation – **N/A**

For peer review only - http://bmjopen.bmj.com/site/about/guidelines.xhtml

Dissemination policy	31a	Plans for investigators and sponsor to communicate trial results to participants, healthcare professionals, the public, and other relevant groups (eg, via publication, reporting in results databases, or other data sharing arrangements), including any publication restrictions – **page 11**
	31b	Authorship eligibility guidelines and any intended use of professional writers – **N/A**
	31c	Plans, if any, for granting public access to the full protocol, participant-level dataset, and statistical code – **N/A**
**Appendices**		
Informed consent materials	32	Model consent form and other related documentation given to participants and authorised surrogates – **N/A**
Biological specimens	33	Plans for collection, laboratory evaluation, and storage of biological specimens for genetic or molecular analysis in the current trial and for future use in ancillary studies, if applicable – **N/A**

*It is strongly recommended that this checklist be read in conjunction with the SPIRIT 2013 Explanation & Elaboration for important clarification on the items. Amendments to the protocol should be tracked and dated. The SPIRIT checklist is copyrighted by the SPIRIT Group under the Creative Commons "Attribution-NonCommercial-NoDerivs 3.0 Unported" license.

For peer review only
